# Supplementary material for: The genome sequence of a phantom cranefly, Ptychoptera contaminata (Linnaeus, 1758)
Source: Wellcome Open Res. 2025 Jun 4;10:308. [Version 1] doi: 10.12688/wellcomeopenres.24276.1 (PMC12355170; doi:10.12688/wellcomeopenres.24276.1)
Supplement: Peer review history

# Reviewer report: Reviewer response for version 1

**DOI:** 10.21956/wellcomeopenres.26774.r125906

**Published:** 2025-08-14

**Authors:** Arun Arumugaperumal

**Affiliations:** 1 Rajalakshmi Engineering College, Chennai, India; 2 Department of Biotechnology, Rajalakshmi Engineering College, Thandalam, Chennai, Tamil Nadu, India

The genome sequence of *Ptychoptera contaminata*, one of the phantom crane flies, is being discussed in this data note. The photograph of the specimen is wonderfully presented as figure 1. The size of the assembly presented is 204.08 Mb spanning 6 chromosomes. In the case of *P. albimana*, which is a related species, there were 7 chromosomes [1]. The authors can comment on this. Consider changing '71 coverage' to '71X coverage'.

Are sufficient details of methods and materials provided to allow replication by others?

Yes

Is the rationale for creating the dataset(s) clearly described?

Yes

Are the datasets clearly presented in a useable and accessible format?

Yes

Are the protocols appropriate and is the work technically sound?

Yes

Reviewer Expertise:

Bioinformatics; Genomics

I confirm that I have read this submission and believe that I have an appropriate level of expertise to confirm that it is of an acceptable scientific standard, however I have significant reservations, as outlined above.

## References

[1] : The genome sequence of a fold-wing cranefly, Ptychoptera albimana (Fabricius, 1787). *Wellcome Open Research* .2024;9: 10.12688/wellcomeopenres.23208.1 10.12688/wellcomeopenres.23208.1

# Reviewer report: Reviewer response for version 1

**DOI:** 10.21956/wellcomeopenres.26774.r125911

**Published:** 2025-07-18

**Authors:** Christopher B. Cunningham

**Affiliations:** 1 University of Georgia, Georgia, USA

The project continues to produce quality resources for the insect genomics community. The methods are sound and relatively well documented. The article needs has enough details to be reasonably reproducible. The data supporting the publication are publicly available.

 Abstract. There is no justification within the abstract, just results. At least a sentence that this was part of a large survey of insects is needed. And one sentence for its possible value.

 Background. Background. The stated motivation for this study in the introduction is fine – part of a very large survey. However, it would be nice for the reader to understand if this is the first of its genus,  is of particular interest for ecological or genetics studies, or something unusual about its biology. Helping the reader understand what this genome might help with investigation in.

 Results. Table 1 is not really needed. They are not details that the reader needs to under the work and they can all be found under the NCBI BioProject ID or are repeated from the methods paragraph.

 BUSCO completeness needs to be assessed with the updated OrthoDB version 12. Difference from 10 to 12 have made a big difference for several insects I work with and colleagues have reported the same. The paper should not be published until this is updated. The new version has been for 6 months.

 Figure 4. Fig 4 has very little information beyond what is found elsewhere in the manuscript and can easily be summarized as a sentence in the results.

 Figure 3 & 5. Suggest lots of assembly dead-ends. I appreciate that they have no hits to anything other than Arthropoda but still the variation in coverage, variation in GC content, and strong Hi-C links among themselves is very odd. It suggests the QC of the genome draft assembly is incomplete.

 The Genome annotation should give the reader some indicator of the quality of the annotation. Nothing is known to the reader other than it’s a reasonable amount of genes. No BUSCO, no Ontology, no percentage of hits to other Dipteran proteins, etc. It was produced by a standing pipeline but the reader does not even have the slightest hint of how well that really worked.

Are sufficient details of methods and materials provided to allow replication by others?

Yes

Is the rationale for creating the dataset(s) clearly described?

Partly

Are the datasets clearly presented in a useable and accessible format?

Yes

Are the protocols appropriate and is the work technically sound?

Yes

Reviewer Expertise:

Genetics, Genomics, Epigenetics, Insects, Behavior, Reproduction.

I confirm that I have read this submission and believe that I have an appropriate level of expertise to confirm that it is of an acceptable scientific standard, however I have significant reservations, as outlined above.

# Reviewer report: Reviewer response for version 1

**DOI:** 10.21956/wellcomeopenres.26774.r125913

**Published:** 2025-07-04

**Authors:** Taro Nakamura

**Affiliations:** 1 National Institute for Basic Biology, Okazaki, Japan

This Data Note reports the chromosomal-level genome assembly of the phantom cranefly, Ptychoptera contaminata, as part of the Darwin Tree of Life project. The work represents a valuable contribution to insect genomics and biodiversity genomics efforts. The manuscript is clear, and provides sufficient methodological detail for reproducibility. The data have been deposited in public repositories, ensuring open access and facilitating downstream research.

 Sequencing was performed using state-of-the-art PacBio HiFi long-read technology and Hi-C scaffolding, yielding a high-quality genome (204.08 Mb, scaffold N50 = 36.52 Mb, 83.64% of the assembly assigned to chromosomes).

 Assembly quality is well-documented using metrics such as BUSCO (93.3% complete), QV (58.3), and k-mer completeness.

 Annotation via Ensembl has identified 11,102 protein-coding genes and 911 non-coding genes.

 The manuscript thoroughly documents the collection, DNA extraction, sequencing, assembly, and annotation protocols, including software versions and quality control procedures.

 Suggestions for Improvement:

 While the inclusion of the Hi-C contact map (Figure 5) is highly valuable, the figure's current presentation—with relatively thick grid lines—makes it difficult to discern finer contact patterns, especially in regions with dense signals. For improved clarity and accessibility, I recommend refining the figure as follows:

 Use thinner grid lines or reduce the opacity of grid overlays so as not to overpower the actual Hi-C signal.

 Conclusion and Recommendation:

 The manuscript provides a high-quality genomic resource with clear, well-documented methods and public data availability. The work meets the standards for a data note, and the minor suggestions above are meant to further enhance clarity and user-friendliness. If the authors can address the suggestions above, this manuscript will be ready for acceptance.

Are sufficient details of methods and materials provided to allow replication by others?

Yes

Is the rationale for creating the dataset(s) clearly described?

Yes

Are the datasets clearly presented in a useable and accessible format?

Yes

Are the protocols appropriate and is the work technically sound?

Yes

Reviewer Expertise:

Developmental Biology

I confirm that I have read this submission and believe that I have an appropriate level of expertise to confirm that it is of an acceptable scientific standard.
